# Bovine tuberculosis: a retrospective study at Jos abattoir, Plateau State, Nigeria

**DOI:** 10.11604/pamj.2016.25.202.5669

**Published:** 2016-11-29

**Authors:** Lilian Akudo Okeke, Olufunmilayo Fawole, Maryam Muhammad, Ikenna Osemeka Okeke, Patrick Nguku, Peter Wasswa, David Dairo, Simeon Cadmus

**Affiliations:** 1 Nigeria Field Epidemiology and Laboratory Training Programme, Abuja, Nigeria; 2 Department of Epidemiology and Medical Statistics, Faculty of Public Health, University of Ibadan, Nigeria; 3 National Veterinary Research Institute, Vom, Plateau State, Nigeria; 4 Federal College of Veterinary and Medical Laboratory Technology, NVRI, Plateau state, Nigeria; 5 African Field Epidemiology Network, Kampala, Uganda; 6 Department of Veterinary Public Health, University of Ibadan, Ibadan, Nigeria

**Keywords:** Bovine tuberculosis, retrospective, cattle, zoonotic, abattoir, Plateau State, Nigeria

## Abstract

**Introduction:**

Nigeria has the thirteenth highest burden of human tuberculosis. The current increasing incidence of tuberculosis in humans, particularly in immune-compromised persons, has given interest in the zoonotic importance of Mycobacterium bovis in developing countries like Nigeria. This study determined the prevalence of bovine tuberculosis as a background information for effective control measures in Plateau State in cattle population.

**Methods:**

We reviewed surveillance records on cattle slaughtered and suggestive tuberculosis lesions from cattle slaughtered annually from 2007-2012 in Jos abattoir, Plateau State. Bovine tuberculosis cases at post mortem were based on examination of characteristics TB lesion on organs by Veterinary officers. We performed descriptive analysis using Epi info version 3.5.3 and Microsoft Excel 2007.

**Results:**

A total of 52, 262 cattle were slaughtered from 2007-2012, out of which 4, 658 (11.2%) had evidence of tuberculosis lesion at post mortem. The average yearly prevalence was 9.1% but varied from a high of 16.3% in 2007 to a low of 3.1% in 2012. Trend analysis showed that bovine tuberculosis had a seasonal variation and peaked mostly in July and August. The number of suggestive Tb lesion cases was highest in the month of August and lowest in the month of January, 2007-2012.

**Conclusion:**

This study shows that bovine tuberculosis is endemic in Plateau State. Trend analysis showed that bovine tuberculosis is seasonal and peaked mostly in July and August. Continuous surveillance through meat inspection is required to prevent zoonotic transmission of bovine tuberculosis.

## Introduction

Bovine tuberculosis results from infection with *Mycobacterium bovis* that occasionally affects other species of mammals. Cattle are the primary host for M. bovis [1]. It is a zoonotic disease that can spread to humans typically by the inhalation of aerosols, ingestion of unpasteurised milk or through breaks in the skin. Raw or undercooked beef can be a source of the infection [2]. In most cases, *M. bovis* is transmitted between cattle in aerosols during close contact. Drinking raw milk is the primary route of *M. bovis* infection in humans; hence human tuberculosis caused by *M. bovis* is mostly extra pulmonary, particularly in cervical lymphadenitis [3]. Although bovine tuberculosis was once found worldwide, control programs have eliminated or nearly eliminated this disease from domesticated animals in countries like Denmark and Sweden [3, 4]. Bovine tuberculosis is still widespread in Africa, parts of Asia and some Middle Eastern countries [5].

In Africa, the occurrence of bovine tuberculosis due to M. bovis in humans is difficult to determine accurately because of technical problems in isolating the microorganism [6]. In West and Central Africa, bovine TB in humans seems to be especially prevalent among the nomadic Fulani tribe [7], who herd their cattle across the borders of the country. Pastoralist use milk which they do not usually boil, from their cattle for food. Bovine TB in humans is becoming increasingly important in developing countries like Nigeria as humans and animals are sharing the same micro-environment and dwelling premises especially in rural areas. There is increasing contact between humans and animals worldwide due to increasing population density and growth especially in poor developing countries where livestock offers important socioeconomic, cultural, and religious pathways out of poverty [8–10]. However, many diseases affect livestock and humans (some of which are zoonoses) with huge negative impact on animal productivity and public health with the poor being particularly vulnerable [11]. Animal and human tuberculosis (TB), emerging or re-emerging and caused by pathogenic bacteria of the Mycobacterium tuberculosis complex, *M. bovis* and *M. tuberculosis* [12] are widespread and affecting the animal industries and human health in Africa [13].

The link between animal and human tuberculosis has long always been known to be strong, as shown by the works of Villemin in 1865 [14] and Koch in 1882 [15], which demonstrated the cross adaptability of the tubercle bacilli from one species to another to cause disease [9]. This was corroborated in 1902 by Ravenel (1902), who demonstrated *Mycobactrium bovis* in a child with tuberculous meningitis.

Nigeria is the most populous country in Africa and it has over 160 million people, with 19.5 million cattle and an unknown population of wildlife ruminants [16]. These factors provide also an opportunity for the easy transmission and spread of bovine type of TB [17]. This is further aggravated by prevalence of BTB, which is estimated between 8.8% and 10.5% Nigeria has the thirteenth highest burden of human tuberculosis among the world’s 22 countries with high Tb burden [17]. Among African countries, Nigeria has the highest estimated number of new cases with nearly 368,000 new cases annually [18]. The situation with animal tuberculosis is less clear, as no national control strategy exists and the degree of zoonotic transmission of tuberculosis from animals to humans is not well known. However, cultural practices exist that could facilitate transmission between cattle and humans [19]. In Plateau State, cultural practices by the nomadic Fulani tribe, who herd their cattle across state boundaries and abattoir workers who slaughter cattle at the abattoir, could facilitate transmission between cattle and humans. This study is a 5 years retrospective study of bovine Tb lesions recorded at the Jos South abattoir. This study was carried out in order to contribute to the knowledge on the epidemiology of the disease which will serve as background information for guidelines for control measures.

## Methods

### Study area

The study was conducted in Plateau State, Nigeria. The state is made up of three agricultural zones; North, Central and South and has seventeen Local Government Areas. The state is located in the North Central geopolitical zone of Nigeria. The vegetation is Guinea savannah and very conducive for livestock, poultry and crop production. Indigenes of Plateau State are mainly engaged in agriculture. The major livestock produced in the state include; cattle (including exotic breeds), sheep, goats and pigs. Majority of the cattle in the state is owned by the Fulani pastoralists, who are widely spread across the state. The abattoir is located at Jos South Local Government Area. It is the only abattoir in the state and serves as a major source of meat and pork, as well as other animal products.

**Study design:** Secondary analysis of abattoir records on annual suggestible bovine tuberculosis lesions from cattle slaughtered at Jos abattoir in Plateau State, Nigeria between the periods of May-June, 2012.

**Study population:** Cattle slaughtered at the Jos abattoir from January, 2007- December, 2012.

**Data collection tools:** A line list was used to obtain information from the abattoir records with respectively to numbers of cattle slaughtered monthly and yearly and numbers of suggestive tuberculosis lesion recorded monthly and yearly.

**Data analysis:** Data was entered and analyzed using Epi info 3.5.3 version software and Microsoft Excel to determine frequencies, proportions and prevalence ratios.

**Ethical consideration:** Since the study is a secondary analysis of existing data, approval from an ethical committee was not needed.

## Results

**Figure 1:**
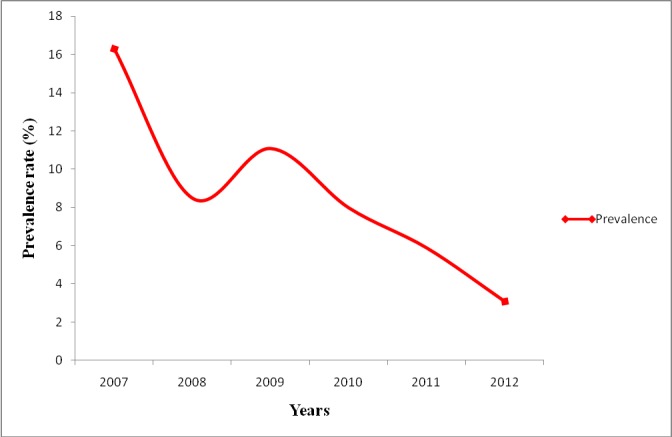
Temporal trend on tubercle lesions from slaughtered cattle at Jos South abattoir, Plateau State, Nigeria, 2007-2012

A total of 51, 262 cattle were slaughtered from 2007-2012. Abattoir records on annual slaughtered cattle and suggestive Tb lesion cases from cattle slaughtered from 2007-2012 reviewed showed that the highest number (16.3%) of tuberculosis lesions in cattle was recorded in 2007 with an overall prevalence rate of 9.1% from 2007-2012 (Table 1). The highest prevalence (16.3%) was in 2007 and the lowest prevalence (3.1) was in 2012 (Figure 1).

**Table 1:** Retrospective abattoir records on suggestive tuberculosis lesions from slaughtered cattle at Jos South Abattoir, Plateau State, Nigeria, 2007-2012

Years	No of Cattle Slaughtered (per year)	Number of suggestive Tb lesions in cattle (per year).	Prevalence rate (%)
2007	6,710	1,091	16.3
2008	12,612	1,068	8.5
2009	11,025	1,224	11.1
2010	10,140	812	8.0
2011	4,931	289	5.9
2012	5,844	178	3.1
**Total**	**51,262**	**4,658**	**9.1^+^**

+ Overall prevalence of Tb lesions in cattle from 2007-2012 is 9.1%

**Figure 2:**
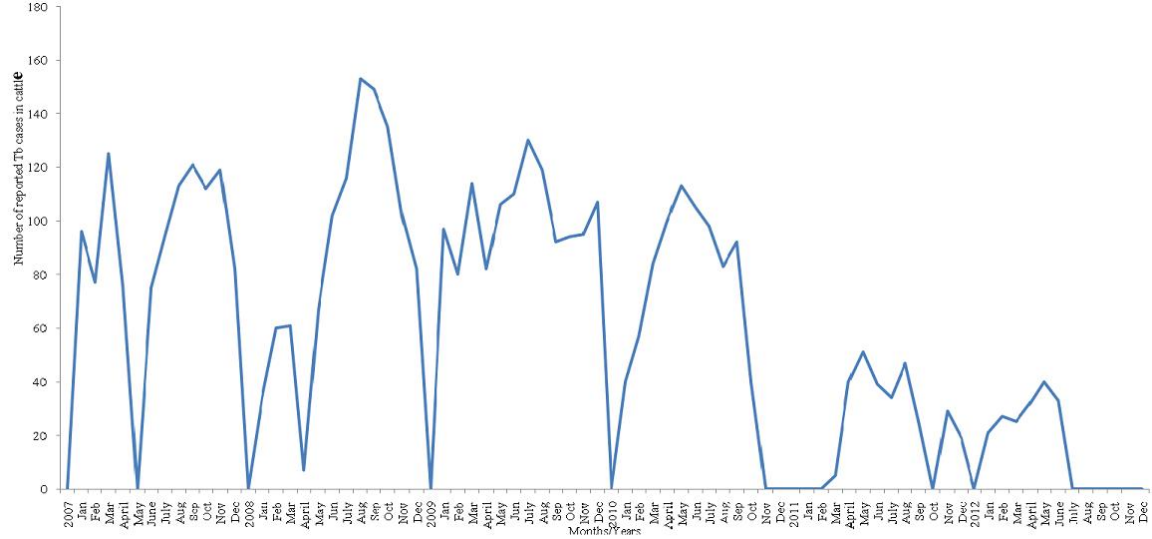
Seasonal trend of annual detection of tubercle lesions in slaughtered cattle at Jos South abattoir, Plateau State, Nigeria, 2007-2012, +Months showing zero were the periods of workers’ strike actions and crisis in Plateau State

**Figure 3:**
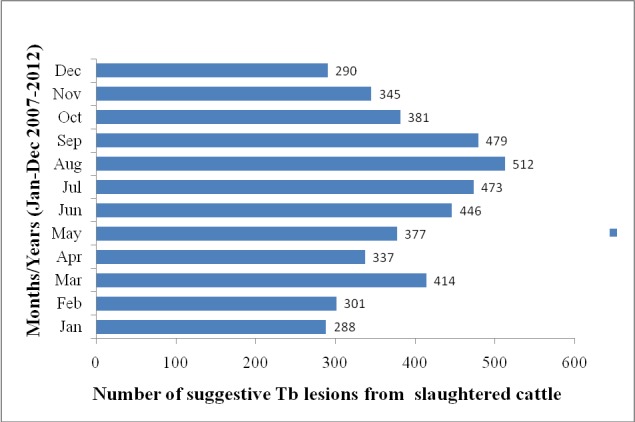
Bar chart showing the number of suggestive Tb lesions from slaughtered cattle at Jos South abattoir, Plateau State, 2007-2012

The number of suggestive Tb lesion cases was highest in the month of August and lowest in the month of January, 2007-2012 (Figure 2). Trend analysis shows number of suggestive TB lesion cases from abattoir record between 2007and 2012. It is of cyclical pattern and peaked mostly in July and August (Figure 3).

## Discussion

Retrospective review of abattoir records on cattle slaughtered and number of suggestive Tb lesion cases recorded annually from 2007-2012 showed the highest prevalence of 16.3% in 2007 and the lowest (3.1%) in 2012. This may have been due to more complete results consequent to the Avian influenza project in 2007, under reporting may have occurred and reduced activities of the abattoir due to religion and ethnic crises in 2012.

Prior to the study period, information on bovine TB in Plateau State was sparse though TB was the most common pathology encountered at abattoir meat inspections. The detection rate of TB lesions was influenced by season but it was higher during stressful periods such as inter-season and peak-season periods and also when slaughtering was elevated during religious feasts and socio-cultural ceremonies. The reason for the wide fluctuation of annual detection rates for the entire period was not clear. Inadequacies in capacity and lack of thoroughness of the veterinary staff carrying out meat inspection could have played major roles. This agrees with [20] who reported that postmortem surveillances for detection of bovine TB lesions in particular depend on the work load, time, and diligence of the inspector conducting the examination. However, it was also not uncommon that when veterinary staff inspect carcasses, condemn and seize infected meat and meat products for disposal, some pathological cases are missed completely due to lack of unassisted command on the part of the veterinary staff over the rough behaviours of butchers and meat trader. Over time and with repeated meat inspections butchers acquire ample knowledge about the nature of pathologies that can lead to condemnation of carcasses just from observing the activities of the veterinary staff. Unruly butchers could obstruct inspection of their animal carcasses or hide lesions from unassisted inspectors. Similar findings have been reported by [21] in neighbouring Nigeria that pathological cases including zoonoses in slaughtered animals were missed due to uncooperative attitudes of butchers in ensuring thorough meat inspection.

Trend analysis of suggestive Tb cases reported from 2007-2012 by months at Jos South abattoir, shows a cyclical pattern and peaked in July and August which shows that it is a seasonal disease, and that season has effect on the rate of transmission of the disease. This is quite different from the findings in the study done by [22] but similar to the observation of [23] in Cross River State abattoir who reported that there is a strong association between the occurrence of tuberculosis lesions and seasonal distribution.

## Conclusion

This study shows that bovine tuberculosis is still prevalent in Plateau state. We therefore, recommend that intensified public education for the awareness about the public health implication of bovine tuberculosis, active surveillance for bovine tuberculosis should be conducted to establish the actual situation of the disease among cattle population and molecular tests will establish the types and species of Mycobacterium causing this lesion because of public health implication of the cases and the role of cattle in the epidemiology of human tuberculosis.

### What is known about this topic

- TB is an airborne infectious disease caused by bacteria, which primarily affects the lungs;
- TB remains a major public health problem worldwide and affects livestock e.g cattle and human health in Nigeria;
- Bovine TB is zoonotic and is known to be caused by Mycobacterium bovis.

### What this study adds

- This study generated epidemiological data on bovine tuberculosis in cattle in Plateau State, through a retrospective survey of Jos South abattoir records for a five-year period (2007 to 2012);
- This study contributed to the knowledge on the epidemiology of the disease which will serve as background information for guidelines for control measures;
- This study provided an information on the prevalence of bovine tuberculosis in Plateau state that could be used in other studies.
